# Tuberin-deficiency downregulates N-cadherin and upregulates vimentin in kidney tumor of TSC patients

**DOI:** 10.18632/oncotarget.2206

**Published:** 2014-07-13

**Authors:** Sitai Liang, Tiffanie Salas, Emre Gencaslan, Baojie Li, Samy L. Habib

**Affiliations:** ^1^ South Texas Veterans Health Care System, San Antonio, TX; ^2^ Akdeniz University Medical School, Antalya, Turkey; ^3^ Bio-X institutes, Key Laboratory for the Genetics of Developmental and Neuropsychiatric Disorders, Ministry of Education, Shanghai Jiao Tong University, Shanghai, China; ^4^ Department of Cellular and Structural Biology, University of Texas Health Science Center at San Antonio and, San Antonio, TX

**Keywords:** N-cadherin, vimentin, Akt, tuberin, mTOR and angiomyolipomas

## Abstract

Angiomyolipomas (AMLs) are associated with cell fibrosis in kidney of Tuberous Sclerosis Complex patients. The mechanism by which the fibrotic proteins accumulated in AMLs has not been explored. In the present study, we investigated the role of Akt/tuberin/mTOR pathway in the regulation cell fibrosis proteins. AML cells that expressed low levels of tuberin showed less expression of N-cadherin and higher of vimentin proteins compared to HEK293 cells. AML cells infected with Ad-tuberin showed a significant decrease in vimentin and an increase in N-cadherin protein expression. In addition, cells treated with rapamycin showed a significant increase in p-Akt and a decrease in p-p70S6K that was associated with a decrease expression of vimentin and a slight increase expression in N-cadherin. On the other hand, cells treated with Akt inhibitor revealed a significant decrease in p-Akt and p-p70S6K that was associated with a significant decrease in vimentin and an increase in N-cadherin expression. In addition, cells transfected with DN-Akt or DN-S6K show significant increase expression in N-cadherin and a decrease in vimentin. Moreover, cells transfected with siRNA against rictor or siRNA against raptor resulted in a decrease in vimentin and an increase N-cadherin expression. Kidney tumors from TSC patients showed significant decrease in N-cadherin and significant increased in vimentin protein expression compared to control kidney tissues. These data comprise the first report to provide the role of Akt/tuberin/mTORC1/2 in the regulation of N-cadherin and vimentin that are involved in the progression of fibrosis in kidney tumor of TSC patients.

## INTRODUCTION

Tuberous Sclerosis complex (TSC) is a genetic disorder, which cause tumors to develop in many organs including kidney. The major phenotype of TSC is multicentric angiomyolipomas rather than renal cell carcinomas (RCCs.) Angiomyolipomas (AMLs) are benign tumors mainly composed of smooth muscle, fat, and blood vessels. AMLs are present in more than 80% of patients with TSC, which is an autosomal dominant tumor suppressor syndrome [1]. On the other hand, kidney cancer is rarely developed in TSC with the occurrence of only 2–3% of all patients in which smooth muscle is often the only component [2-4]. The smooth muscle cells of angiomyolipomas are deriving from a precursor termed as the perivascular epithelioid cell (PEC) [5]. The typical features of PEC-derived lesions are the dual expression of smooth muscle and melanocytes [6]. AMLs most commonly occur in the kidneys and are often asymptomatic, however, enlargement and bleeding could occur leading to hemorrhage and renal impairment [7-9]. Hereditary forms of renal angiomyolipoma tend to be larger, bilateral, multiple and manifest at a younger age compared with sporadic forms [10].

The loss of heterozygosity (LOH) at the loci of either TSC1 or TSC2 has been reported many times in TSC-associated hamartomas as well as in sporadic tumors of non-TSC patients [11,12]. Tuberin is a tumor suppressor protein that produced by TSC2 gene. Absence or inactivation of TSC gene is reported to be associated with several defects such as abnormal cellular migration, proliferation, and differentiation [13-16]. The expression of tuberin is induced by acute renal injury, which indicates it may be functioning as an acute-phase response gene, limiting the proliferative response after injury [17].

AMLs are associated with high proliferation of fibrous tissue in kidney of TSC patients but the mechanisms are not fully understood so far. Several studies showed that the mammalian target of rapamycin (mTOR) serves a critical role in regulating the translational machinery that affects growth, proliferation, and differentiation, all of which are abnormally manifested in TSC lesions [18,19], which are associated with accumulation of fibrous proteins. Cadherins functioning as calcium-dependent are a class of type-1 transmembrane proteins that play an important role in cell adhesion by forming adherens junctions to bind cells and tissues together. Cells lacking tuberin because of inactivation of the *TSC2* gene fail to localize polycystin-1 and E-cadherin appropriately to these junctions [20]. N-cadherin and E-cadherin are the two major components of cadherin family with similar functions. No published data so far demonstrate the expression of major fibrosis following proteins, N-cadherin and vimentin in kidney AMLs.

The mechanisms by which tuberin regulates N-cadherin and vimentin proteins has not been explored. In the present study, we investigated the role of tuberin/mTOR in regulating N-cadherin and vimentin as major fibrosis proteins involved in the progression and development of angiomyolipomas in TSC patients.

## RESULTS

### AML cells expresses less of N-cadherin and higher of vimentin proteins

**Figure 1:**
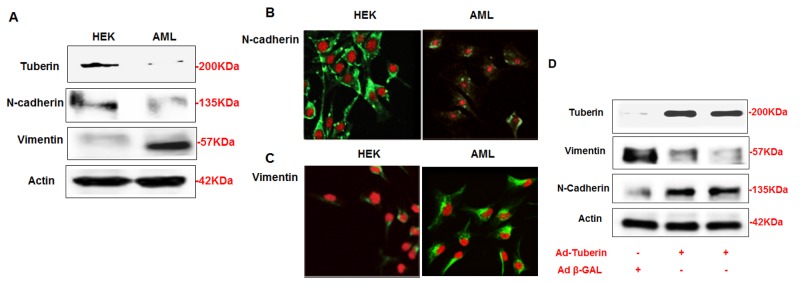
AML cells express less of N-cadherin and higher of vimentin proteins compared to HEK293 cells (A) Cells lysates from AML and HEK293 cells were subjected Western blot analysis. Immunoblot analysis show increased in vimentin and decreased in N-cadherin protein expression in AML cells compared to HEK293 cells. (B) AML and HEK293 cells were immunostained for vimentin and N-cadherin using double fluorescence labeling method. The cells were incubated with rabbit antibody against vimentin or N-cadherin followed by secondary anti-rabbit IgG conjugated with FITC. The cells were reacted with Vectashield Mounting Medium with Propedium Iodide (PI) for nuclear staining. (B&C) FITC green signals for N-cadherin and vimentin were detected using a filter with excitation range of 488 nm and PI red signals for nuclear DNA using a filter with excitation at 535 nm. Overlay of vimentin or N-cadherin and DNA staining, demonstrating cell membrane staining for N-cadherin and cytoplasmic staining for vimentin in AML cells. To show staining specificity, control cells were stained without primary antibody. (D) Upregulation of tuberin resulted in decrease in vimentin and increase N-cadherin expression in AML cells. AML cells were infected with adenovirus 6.01 expressing tuberin complementary DNA. An adenovirus vector expressing protein (Adb-GAL) was used as a control. Immunoblot analysis shows overexpression of tuberin decreases vimentin and increases N-cadherin protein expression. Actin was used as a loading control.

To determine the expression of N-cadherin and vimentin in AMLs, AML cells and HEK293 cells were seeded without any treatment and harvested 48 hrs later. Protein from AML cells and HEK293 cells were extracted and subjected to Western blot. AML cells showed decreased expression of tuberin, N-cadherin, and higher expression of vimentin compared to HEK293 cells (Figure 1A). Immunofluorescence staining was demonstrated to confirm the expression of N-cadherin and vimentin in both cells. The staining clearly showed decrease in the expression of N-cadherin in AML cells compared to strong staining was detected in HEK293 cells (Fig. 1B). On the other hand, AML cells showed stronger staining of vimintin compared to a weak staining in HEK293 cells (Fig. 1C). This data is consistent with the result of Western blot. Together, these data suggest that tuberin positively regulates N-cadherin and negatively regulates vimentin in normal cells.

### Tuberin regulates N-cadherin and vimentin expression

In order to confirm our hypothesis that tuberin regulates the expression of N-cadherin and vimentin in AML cells, AML cells were infected with adenovirus 6.01 expressing tuberin complementary DNA (C-DNA). Cells were harvested after 48 hrs of infection and proteins were extracted and subjected to Western blot. An adenovirus vector expressing protein (Ad b-GAL) was used as a control. Western blot showed that the overexpression of tuberin decreased the expression of vimentin and increased the expression of N-cadherin (Figure 1D). This data indicated that tuberin is an upstream regulator of both N-cadherin and vimentin in AML cells.

### Rapamycin treatment decreased vimentin and N-cadherin via Akt/pS6K pathway in AML cells

**Figure 2:**
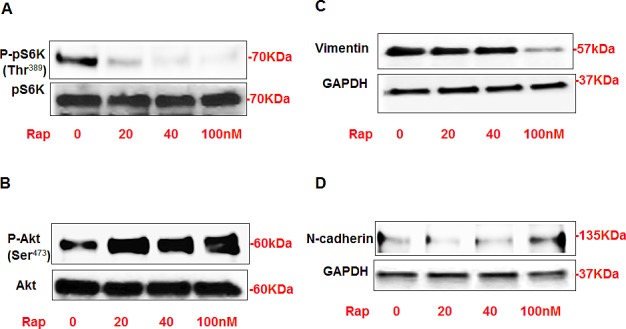
Rapamycin treatment significantly decreased P-p70S6K at Thr^389^ and increased p-Akt at Ser^473^ that associated with decreased vimentin and increased N-cadherin expression in AML cells AML cells were treated with different concentrations of rapamycin (0-100nM) for 24h. Western blot analysis was performed in cell lysates using p-p70S6K, p70S6k, p-Akt, Akt, vimentin, N-cadherin and GAPDH antibodies. Cells treated with rapamycin showed significant decrease in protein expression of (A) p-p70S6K and vimentin. On the other hand, significant increase in (B) p-Akt expression and slight increase in (D) N-cadherin showed in cells treated with rapamycin compared to non-treated cells. GAPDH was used as a loading control.

Our previous published data demonstrated that rapamycin treatment played a role in regulation of fibronectin in AML cells. To determine whether rapamycin treatment also have any effect on other cell fibrosis of AMLs, AML cells were treated with different concentrations of rapamycin (0-100nM) for 24 hrs. Protein from rapamycin treated and untreated cells were extracted and subjected to Western blot analysis. Blots were incubated with p-p70S6K, p70S6k, p-Akt, Akt, vimentin, and N-cadherin antibodies. AML cells treated with rapamycin showed significant decreased expression of p-p70S6K, significant decreased in vimentin at higher dose (100nM) and slightly increased expression of N-cadherin (100nM) (Fig. 2A,C,D). On the other hand, the expression of p-Akt was significantly increased (Fig. 2D). Either increased expression of N-cadherin or decreased vimentin is contingent on the dosage of rapamycin. These data indicate that rapamycin treatment plays a role in the fibrosis of AML via P-p70S6K and/or P-Akt and the effects are rapamycin dose-dependent.

### Inhibition of Akt significantly decreased the expression of vimentin and increased the expression of N-cadherin in AML cells

**Figure 3:**
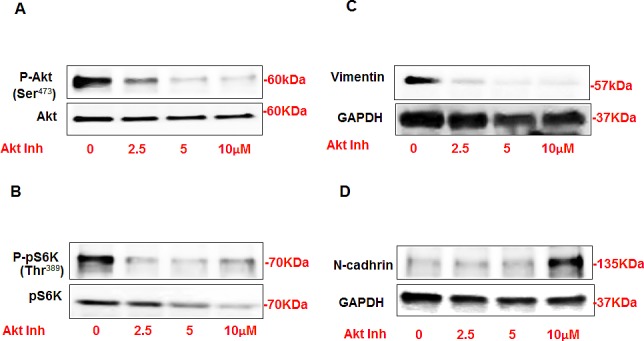
Inhibition of Akt resulted in significant decrease in vimentin and increase in N-cadherin expression in AML cells AML cells were treated with different concentrations of Akt inhibitor IV (0-10μM) for 24h. Western blot analysis was performed in cell lysates using p-Akt, Akt, p-p70S6K, p70S6k, vimentin, N-cadherin and GAPDH antibodies. Cells treated with Akt inhibitor showed significant decrease in (A) P-Akt at Ser^473^ and (B) p-p70S6K at Thr^389^ compared to non-treated cells. Decrease in Akt phosphorylation is associated with significant decrease in (C) vimentin expression in cells treated with lower dose 2.5μM of Akt inhibitor. (D) Cells treated with lower dose of Akt inhibitor (2.5-5 μM) did not show any alteration in N-cadherin while cells treated with higher dose (10 μM) showed significant increase in N-cadherin expression compared to no-treated cells. GAPDH was used as a loading control.

In order to confirm the role of Akt in regulating the expression of N-cadherin and vimentin, AML cells were treated with different concentrations of Akt inhibitor IV (0-10μM) for 24 hrs. Protein was extracted and Western blot analysis was performed and blotted against p-Akt (Ser^473^), Akt, p-p70S6K (Thr^389^), p70S6k, vimentin, N-cadherin antibodies. AML cells treated with Akt inhibitor showed significant decrease in P-Akt at Ser^473^ (Fig. 3A) and p-p70S6K at Thr^389^ (Fig. 3B) compared to non-treated cells. The expression of vimentin was also significantly decreased at very low doses of Akt inhibitor (2.5μM) and abolished at higher dose (5-10μm) (Figure 3C). AML cells treated with lower doses of (2.5-5μM) did not show any changes in N-cadherin expression while cells treated with higher dose (10μM) showed significant increase in N-cadherin expression compared to non-treated cells (Figure 3D). These data indicated that the change in both protein expressions is dose-dependent for Akt inhibitor. Together, these data indicates that Akt appositely regulates the expression of N-cadherin and vimentin in AML cells.

### Akt and mTOR regulate N-cadherin and vimentin in AML cells

**Figure 4:**
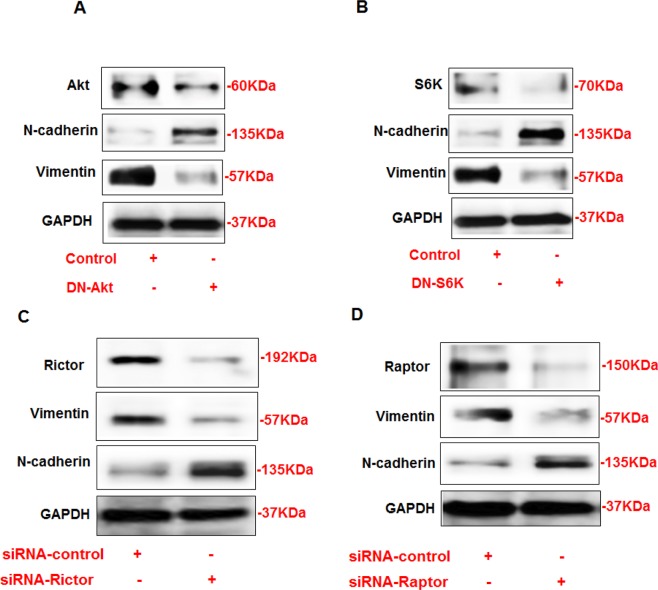
Opposite regulation of N-cadherin and vimentin by DN-Akt and DN-S6K in AML cells (A) Immunoblot analysis shows that downregulation of Akt by DN-Akt results in an increased in N-cadherin and decreased in vimentin protein expression in AML. In addition, downregulation of S6K by DN-S6K results in decreased in vimentin and increased in N-cadherin protein expression in AML cells. Rictor and Raptor regulates N-cadherin and vimentin (C) Immunoblot analysis shows that downregulation of rictor by siRNA directed against rictor results in decreased in vimentin expression and increased in N-cadherin protein expression in AML cells. In addition, downregulation of raptor by siRNA directed against raptor (D) results in decreased in vimentin expression and increased in N-cadherin protein expression in AML cells. GAPDH was used as a loading control.

To further investigate the mechanism by which Akt and mTOR regulate N-cadherin and vimentin expressions, AML cells were transfected with dominant negative (DN) of Akt and DN-p70S6K. The cells were harvested 24 hrs later after transfection and proteins were extracted. Immunoblot analysis showed that the downregulation of Akt by DN-Akt increased the expression of N-cadherin and decreased the expression of vimentin in AML cells (Figure 4A). In addition, the downregulation of p70S6K by DN-p70S6K resulted in the decreased expression of vimentin and increased expression of N-cadherin in AML cells (Figure 4B). These data further confirmed that both Akt and p70S6K are major kinases regulate of N-cadherin and vimentin expression.

### Rictor and Raptor oppositely regulate N-cadherin and vimentin

To determine the mechanism by which mTORC1 or mTORC2 plays a role in regulation of N-cadherin and vimentin, AML cells were transfected with either siRNA against Rictor or siRNA against Raptor or control siRNA. The AML cells were harvested after 24 hrs of transfection. Immunoblot analysis showed that the downregulation of rictor by siRNA directed against mTORC2 resulted in the decreased expression of vimentin and increased expression of N-cadherin in AML cells (Figure 4C). Additionally, the downregulation of raptor by siRNA directed against mTORC1 also resulted in the decreased expression of vimentin and the increased expression of N-cadherin in AML cells (Figure 4D). These data indicate that both mTORC1 and mTORC2 has a strong effect on regulation of N-cadherin and vimentin expressions in AML cells.

### Tuberin deficiency upregulates vimentin expression in kidney tumor of TSC patients

**Figure 5:**
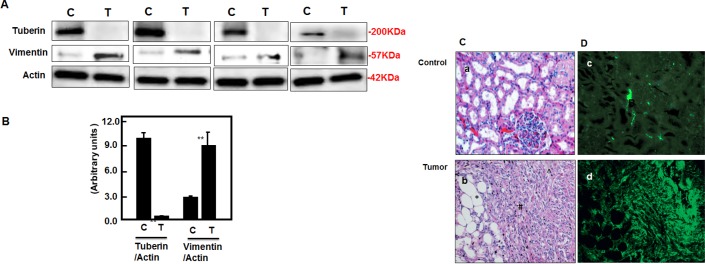
Deficiency of tuberin results in increased vimentin protein expression in tumor kidney tissue of TSC patients (A) Representative Immunoblot analysis showed significant decreased in tuberin and increased in vimentin protein expression in tumor kidney (T) from patients with tuberous sclerosis compared to normal kidney tissues. Actin was used as loading control. (B) Histograms represent means±SE (n=6). Significant difference from control is indicated by **P<0.01. (C) H&E staining shows (a) a normal tubular and glomerular structure in control kidney tissue and (b) *fat, ^vessel and # smooth muscle cells types in kidney angiomyolipoma tissue of TSC patients. (D) Kidney sections were stained with vimentin followed by immunofluroscene staining. Control of kidney (c) shows a few cells stained with vimentin while (d) most of blood vessel and smooth muscle cells were stained in kidney tumor tissue. Control sections in both procedures were incubated without primary antibody.

To determine whether deficiency of tuberin in tumor tissue of TSC patients has an effect on vimentin protein expression, we extracted proteins from tumor kidney tissues of TSC patients and normal kidney tissues of healthy subjects. Western blot analysis were performed and blotted against tuberin and vimentin antibodies. Representative blots showed significant decreased expression of tuberin is associated with significant increase in vimentin expression in tumor kidney of TSC patients (Figure 5A&B). On the other hand, weak vimentin expression was detected in healthy subject of normal kidney tissues (Figure 5A&B). H&E staining showed normal tubular and glomerular structure in control kidney tissues, while fat, vessel and smooth muscle cells were shown in kidney section of angiomyolipoma tissue (Figure 5Ca&b). To confirm the expression of vimentin in kidney tissues from normal and tumor samples, immunofluorescence staining was performed. Data in Figure 5Dd showed a strong staining of vimentin in vessel and smooth muscle cells as well as around the fat cells in kidney section of angiomyolipoma tissue. While weak staining of vimentin was detected in normal tubular cells of kidney section of healthy subject (Figure 5Dc).

### Tuberin deficiency downregulates N-cadherin expression in kidney tumor of TSC patients

**Figure 6:**
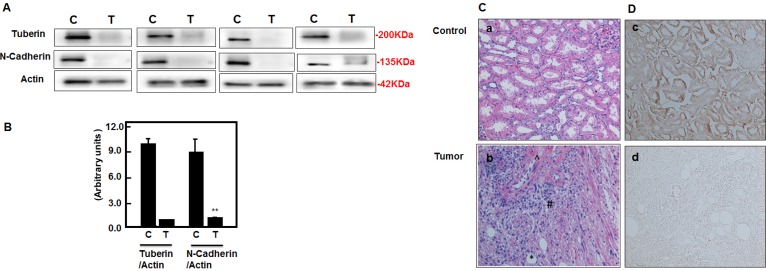
Deficiency of tuberin results in decreased N-cadherin protein expression in tumor kidney tissue of TSC patients (A) Representative Immunoblot analysis showed significant decreased in tuberin and decreased in N-cadherin protein expression in tumor kidney (T) from patients with tuberous sclerosis compared to normal kidney tissues. Actin was used as loading control. (B) Histograms represent means±SE (n=6). Significant difference from control is indicated by **P<0.01. (C) H&E staining shows (a) a normal tubular and glomerular structure in control kidney tissue and (b) *fat, ^vessel and # smooth muscle cells types in kidney angiomyolipoma tissue of TSC patients. (D) Kidney sections were stained with N-cadherin followed by horseradish peroxidase staining. Control of kidney (c) shows a few cells stained with N-cadherin while (d) most of blood vessel and smooth muscle cells were stained in kidney tumor tissue. Control sections in both procedures were incubated without primary antibody.

To determine whether deficiency of tuberin in tumor tissue of TSC patients have effect on N-cadherin protein expression, Western blot analysis were performed and blotted against tuberin and N-cadherin antibodies. Representative blots showed significant decreased expression of tuberin is associated with significant decrease in N-cadherin expression in tumor kidney of TSC patients (Figure 6A&B). On the other hand, strong N-cadherin expression was significantly detected in healthy subject of normal kidney tissues (Figure 6A&B). H&E staining showed normal tubular and glomerular structure in kidney section of control subjects while fat, vessel and smooth muscle cells were shown in kidney section of angiomyolipoma tissue from TSC patients (Figure 6Ca&b). To confirm the expression of N-cadherin in normal kidney tissues and tumor samples, immunoperoxidase staining was demonstrated. N-cadherin staining showed significant decrease in whole tumor kidney tissue sections compared to kidney section of normal tissue (Figure 6Dc&d). Together, these data show that tuberin is a key molecule in regulating cell fibrosis such as N-cadherin and vimentin in kidney tumor of TSC patients.

**Figure 7:**
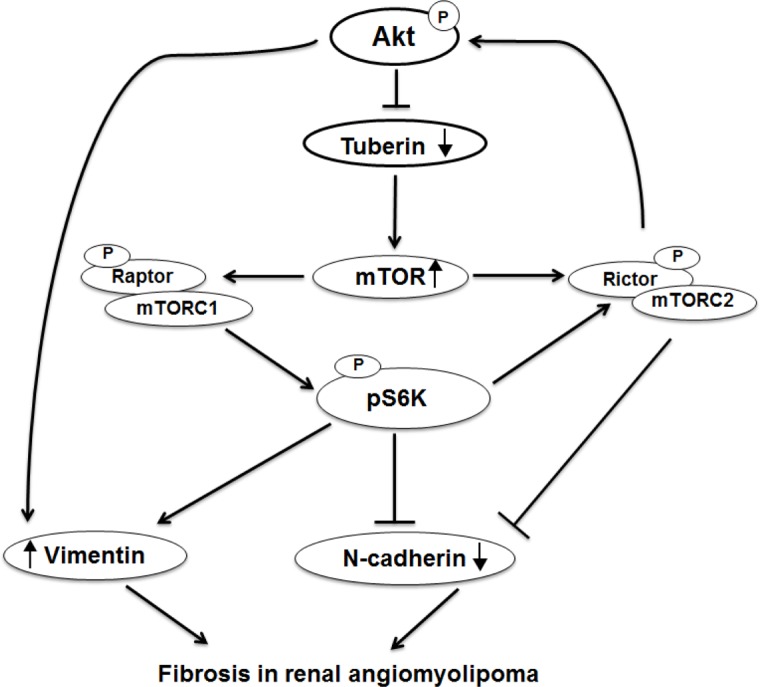
Proposed model of regulation of N-cadherin and vimentin in kidney angiomyolipoma Deficiency in tuberin upregulates vimentin and downregulates N-cadherin in kidney tumor of TSC patients. In addition, activation of Akt enhances vimnetin and decreases N-cadherin expression while activation of mTORC1/2 increases vimentin and decreases N-cadherin resulted in cell fibrosis progression in renal angiomyolipoma of tuberous sclerosis patients.

## DISCUSSION

This study provides the first evidence that tuberin oppositely regulates the expressions of N-cadherin and vimentin in AML renal cells as well as in kidney angiomyolipomas of TSC patients. These data indicate a novel role for tuberin in the regulation of cell fibrosis proteins and provide a potential mechanism by which TSC2 mutation predisposes to the genesis and progression of fibrosis in the kidney angiomyolipomas of TSC patients. Several approaches were utilized to conclusively demonstrate that Akt/tuberin/mTOR pathway involve in regulation of N-cadherin and vimentin proteins. First, very weak staining of N-cadherin and strong staining of vimentin were detected in AML cells compared to HEK293 cells by immunofluorescence staining and Western blot analysis. Second, overexpression of tuberin was associated with downregulation of vimentin and upregulation of N-cadherin in AML cells. Third, inhibition of mTOR by rapamycin inhibits the expression of vimentin by blocking mTORC1, but enhances the expression of N-cadherin at high concentrations. Fourth, inhibition of Akt activation nearly abolished the expression of vimentin and significantly stimulated the expression of N-cadherin at high dosages. Fifth, downregulation of either Akt or P70S6K in AML cells by DN-DNA plasmid correspondingly promotes expression of N-cadherin and inhibits the expression of vimentin in AML cells. Sixth, siRNA against rictor or raptor transfected into the AML cells resulted in downregulation of vimentin and increased N-cadherin protein expression. Additionally, Western blot analysis and immunofluorescence/immunoperoxidase staining showed that the deficiency of tuberin is associated with decreased expression N-cadherin and increased expression of vimentin in tumor tissues compared to control kidney tissue. Altogether, these data suggest that Akt/tuberin/mTOR pathway plays a role in the regulation of fibrosis in kidney angiomyolipomas of TSC patients.

The classic cadherins (E-cadherin, N-cadherin, and P-cadherin) are transmembrane adhesion glycoproteins, which link to the actin cytoskeleton by different catenins [21]. E-cadherin is a calcium-dependent cell-cell adhesion molecule that is repressed in epithelial to mesenchymal transition (EMT) occurring in carcinomas. Previous studies highlighted the importance of epithelial to mesenchymal transition in prostate cancer and demonstrated that a switch from E-cadherin to N-cadherin expression is important in the progression of prostate cancer [22]. Our data showed a weak staining of N-cadherin in AML cells while strong staining was detected in the cytoplasm and cell membrane in normal renal cells (HEK293) suggesting N-cadherin may serve as an additional diagnostic marker for angiomyolipoma of TSC patients. One of the critical steps driving EMT is the repression of E-cadherin, resulting in loss of cell-cell adhesion. E-cadherin is expressed in most epithelial cells in which adherens junctions are formed to create the multicellular organization important for the formation and maintenance of bodily compartments [23]. Recent studies provide evidence that the localization of E-cadherin membranes are regulated by the Akt/mTORC1 pathways as a result of lossing *TSC2* and that lead to a significant reduction in membrane E-cadherin. Consequently, cells deficient in TSC2 are less adhesive and more prone to detach and to undergo EMT [24]. In addition immunestaining of E-cadherin showed that most cases of angiomyolipoma (98%) were positively stained for E-cadherin [25]. To our knowledge, there is no published data so far about expression of N-cadherin in kidney angiomyolipoma of TSC patients.

Cadherin is an important protein in cell-cell adhesion. Loss of cadherins is believed to be the first key step in development of tubulointerstitial fibrosis and EMT [26–28]. The decrease of cell membrane N-cadherin staining in the AML cells is consistent with the previous observations of mislocalization of E-cadherin in TSC2^−/−^ cells [24] suggesting that these trafficking proteins are defective because of the loss of *TSC2* function. Development of cell fibrosis is the result of activation and differentiation of fibroblasts, which secret extracellular matrix proteins within the tissues. Recent studies also support the importance of loss of proximal tubular-specific N-cadherin in promotion of tubulointerstitial fibrosis [27]. Our data show that deficiency in tuberin in AML cells resulted in increased expression of vimentin and decreased expression of N-cadherin suggesting that tuberin is a potential molecule involved in the development of fibrosis in angiomyolipomas. In addition, re-introducing tuberin into AML cells resulted in decreased expression of vimentin and increased expression of N-cadherin confirmed the role of tuberin in regulating both fibrosis proteins.

Despite extensive characterization of the *TSC2* pathway in the regulation of mTOR and other downstream signals, the molecular mechanisms that link the biochemical pathway that involve in developing cell fibrosis in TSC patients with angiomyolipoma remain poorly understood. Our recent published data showed that AML cells and kidney angiomyolipoma tissues from TSC patients expressed higher levels of αSMA compared to normal cells and control kidney tissues [13]. Here, we showed that treatment AML cells with rapamycin significantly decreased expression of vimentin and N-cadherin confirming the role of mTOR in regulation of both proteins. The regulation of vimentin and N-cadherin via inhibiting mTORC1/C2 was confirmed by using siRNA-raptor and siRNA-rictor. These data show that both mTORC1 and mTORC2 are involved and function equally to regulate N-cadherin and vimentin in AML cells. Consistent with our data, previous studies showed that increased mTOR/S6K1 signaling enhanced loss of adherens junction markers and increased fibrosis [29,30]. Furthermore, our data showed that activation of Akt (measured by phosphorylation of Akt at Ser^473^) and activation of mTORC1 (measured by phosphorylation of p70S6K at Thr^389^) are involved in regulation of N-cadherin and vimentin in AMLs. In addition, we show that downregulation of either P-Akt or P-p70S6K significantly upregulated N-cadherin and downregulated vimentin protein expression in AML cells. Recent studies show that Ang II activation of the AT1R contributes to proximal tubular (PT) brush border injury and remodeling, in part, due to enhanced mTOR/S6K1 signaling which promotes tubulointerstitial fibrosis through loss of N-cadherin [31]. Contemporaneous with these findings, there were associated reductions in the PT-specific adhesion molecule N-cadherin and ultrastructural findings of PT remodeling consistent with EMT and tubulointerstitial fibrosis [31].

Downregulation of tuberin resulted in decreased expression of N-cadherin and increased expression of vimentin in tumor tissues of TSC-patient kidneys. This is consistent with the published data from the studies in models of diabetic and polycystic kidney disease suggested that inhibition in mTOR activity slowed down the progression of tubulointerstitial fibrosis [29, 32, 33]. In addition, the immunofluorescence staining data confirmed the decreased expression of N-cadherin and increased expression of vimentin in kidney tumor tissues of TSC patients (Figure 5 & 6). These data indicate that tuberin regulates progression of fibrosis in kidney tumor tissues. A case study showed that treatment of TSC patients with low dose of rapamycin reduced the facial angiofibromas, renal AML volume, improved of blood pressure control and absent bleeding over 12 months of treatment suggesting the role of rapamycin as an anti-fibrotic and anti-proliferative drug [34]. Our data showed the downregulation of vimentin and upregulation of N-cadherin in AML cells treated with rapamycin supported the role of mTOR in regulating both fibrosis proteins. These data elucidate our understanding of the effect of rapamycin on regulating of cell adhesion protein that are involved in tubulointerstitial fibrosis and progression of angiomyolipomas.

In conclusions, these data describe a novel role of tuberin in the regulation of cell fibrosis proteins. We show that tuberin deficiency is associated with increased expression of fibrosis protein (vimentin) and decreased expression of cell adhesion protein (N-cadherin) in kidney tumor tissue of patients with TSC. Our data confirmed the important role of Akt/tuberin/mTORC1/C2 pathway in the regulation of both proteins using several approaches. Our findings highlight an important role of tuberin in regulating cell fibrosis that contributes in the pathogenesis of kidney angiomyolipoma in TSC patients. Collectively these data provide the mechanism whereby tuberin might inhibit the formation of fibrosis and progression of tumorigenesis.

## MATERIALS AND METHODS

Kidney angiomyolipoma tissues from TSC patients with renal angiomyolipoma (total of 6) and unrelated healthy people (total of 6) were obtained from the Brain and Tissue Bank for Development Disorders (University of Maryland, Baltimore, Maryland, USA). The study has been ethically approved by the Institutional Review Board of The University of Texas Health Science Center at San Antonio, TX.

### Cell Culture

Angiomyolipoma (AML) cells derived from human kidney of TSC patient were generously provided by Dr. Elizabeth Henske (Harvard Medical School, MA) (13). The cells were grown in DMEM supplemented with 10% FBS. Human embryonic kidney epithelial cells (HEK293) were obtained from American Type Culture Collection (Manassas, VA) and maintained in DMEM with 10% FBS. All cell lines were grown at 37°C in a humidified atmosphere of 5% CO_2_.

### Rapamycin and Akt inhibitor treatments

AML cells were grown in to 70–80% confluence then quiescent by serum deprivation overnight before treatment. Cells were treated with serial concentrations of Akt inhibitor IV (0-10 μM) or serial concentrations of rapamycin (0-100nM). The cells were then incubated for 24 hrs at 37°C in a humidified atmosphere of 5% CO_2_ before harvested for western blot analysis.

### Overexpression of tuberin

AML cells were grown to 30–50% confluence, made quiescent by serum deprivation for 24 hrs and then infected at room temperature for 1h with adenovirus 6.01 carrying the TSC2 gene [35]. Viral stocks were prepared and tittered using the serial dilution technique as described in the Adeno-X Expression Systems User Manual (Clontech Laboratories). Infection of cells with 20 multiplicity of infection (MOI) showed appreciable expression of TSC2 protein. An adenovirus vector expressing protein (Adβ-GAL) was used as a control. The cells were then incubated for 48 hrs at 37°C in a humidified atmosphere of 5% CO_2_. Cells were washed twice with PBS buffer and western blot analysis was performed on the cell extracts as above using tuberin, vimentin and N-cadherin antibodies.

### Immunofluorescence staining of vimentin and N-cadherin in AML cells

A double fluorescence labeling method was used as described previously with minor modifications [36]. The cells were washed with PBS, fixed, and incubated with rabbit antibody against vimentin or N-cadherin (Cell Signaling Technology, MA), followed by secondary anti-rabbit IgG conjugated with FITC. The cells were reacted with Vectashield Mounting Medium with Propedium Iodide (PI) (Vector Laboratories). In this assay, DNA was labeled with PI, and vimentin or N-cadherin was identified by the primary monoclonal antibody FITC green signals. FITC green signals for vimentin or N-cadherin was detected using a filter with excitation range 450–490 nm and PI red signals for nuclear DNA using a filter with excitation at 535 nm. FITC and propidium iodide were detected using an Olympus Research microscope equipped for epifluorescence with excitation and band pass filters. To show staining specificity, control cells were stained without primary antibody.

### siRNAs of raptor and rictor

AML cells were grown in six-well plates. Prior to transfection, cells (30–50% confluent) were washed with PBS and media was replaced with 800 μl of OPTI-MEM (Invitrogen, Carlsbad, CA). In parallel, 4 μl of oligofectamine (Invitrogen, NY) were combined with 11 μl of OPTI-MEM I and incubated at room temperature for 10 min. Raptor and rictor siRNA kits were purchased from Santa Cruz Biotech (Santa Cruz, CA). The indicated duplex of 1.5 μg were diluted into 180 μl of OPTI-MEM I, added to the Oligofectamine/OPTI-MEM I mixture and incubated at room temperature for 20 min. The siRNA complexes were then added to the cells. After incubation for 3–4 hrs in a 5% CO_2_ incubator, 1 ml of fresh medium was added to a final serum concentration of 10%. Forty-eight hours after transfection, cells were harvested for western blot analysis. The control construct used in parallel experiments contains four, pooled, non-specific siRNA duplexes provided with the kits.

### Western analysis

Homogenates of kidney cortex or cell lysates were prepared as described previously [37]. Protein concentrations were determined with the Bradford assay using bovine serum albumin as a standard [38]. Western blot analysis was performed as described previously [39]. Tuberin, phospho-p70S6K, p70S6K, p-Akt, Akt, vimentin, N-cadherin, rictor and raptor antibodies were from Cell Signaling (Boston, MA); GADPH and antibody was obtained from Santa Cruz Biotechnology (Santa Cruz, CA) and Actin antibody from Calbiochem (Billerica, MA). An enhanced chemiluminescence kit (Amersham, NJ) was used to identify protein expression. Expression of each protein was quantified by densitometry using National Institutes of Health image 1.62 software and normalized to a loading control.

### Immunoperoxidase staining of N-cadherin in angiomyolipoma tissues

Detection of N-cadherin was performed on paraffin sections of normal and tumor kidney by immunoperoxidase histochemical staining [39]. Kidney sections underwent a protease digestion step before they were incubated with rabbit anti-N-cadherin antibody (Cell Signaling, Boston, MA) for 30 min then washed twice with PBS. Sections were then incubated with horseradish peroxidase labeled anti-rabbit antibody for 30 min. The horseradish peroxidase was developed with diaminobenzidine tetrahydrochloride and hydrogen peroxide in PBS. Control sections in both procedures were incubated without primary antibody.

### Immunofluorescence staining of vimentin in angiomyolipoma tissues

Fluorescence labeling method was used as described previously with minor modifications [36]. The cells were washed with PBS, fixed, and incubated with rabbit antibody against vimentin (Cell Signaling, MA), followed by secondary anti-rabbit IgG conjugated with FITC green signals. FITC green signals was detected using a filter with excitation range 450–490 nm using an Olympus Research microscope equipped for epifluorescence with excitation and band pass filters. To show staining specificity, control cells were stained without primary antibody.

### Statistics

Data are presented as mean ± standard error. Statistical differences were determined using ANOVA followed by Student Dunnett's (Exp. vs. Control) test using 1 trial analysis. *P-*values less than 0.05 and 0.01 were considered statistically significant.
